# Metabolic abnormalities in obese children attending a tertiary care centre in South India

**DOI:** 10.1186/1687-9856-2013-S1-P104

**Published:** 2013-10-03

**Authors:** SM Zechariah, L Priyambada, A Simon

**Affiliations:** 1 Division of Pediatric Endocrinology, Department of Paediatrics, Christian Medical College, Vellore, Tamilnadu India

## Aims

To describe the profile of metabolic abnormalities in obese and overweight children presenting to Paediatric Endocrinology Clinic.

## Methods

This is a prospective descriptive study of 80 children and adolescents, between 5.0-16.0 years of age, who were obese or overweight attending the Pediatric Endocrinology Clinic of the Christian Medical College, Vellore, S India during October 2011 to February 2012.

All children with syndromic obesity, hypopituitarism, hypothyroidism, familial hyperlipidemia, and on steroids or on pharmacotherapy for obesity were excluded from the study. Body weight (kg), standing height (cm), waist circumference (cm), hip circumference (cm) and repeated blood pressures were measured in all subjects. Blood investigations included fasting glucose and 2 hour post prandial glucose levels, fasting insulin levels, HbA1C levels and fasting lipid profile.

## Results

80 children and adolescents (females n=33, males n=47) were recruited; 65% in the obese category, 15% were overweight and 20% were morbidly obese. The mean age in males was 11.32 ± 2.605 yrs and in females was 10.85 ± 2.970 yrs. The mean waist circumference among the pubertal children was 97.13 cm ± 10.913 and 88.75% of the subjects had waist circumference more than 90th centile for age and sex.

Dyslipidemia was the most prevalent metabolic abnormality detected. Insulin resistance was present in more than a fourth of our children, with a higher incidence among girls. Comparison between boys and girls showed that boys had a higher prevalence of decreased HDL (46.81% vs 33.33%) and increased triglycerides (51.06% vs 27.27%). Those who had attained puberty had higher prevalence of decreased HDL (42.86% vs 40%) and hypertension (11.42% vs 6.66%). Among the groups, prepubertal boys had the highest prevalence of dyslipidemia, both elevated triglycerides and decreased HDL. This has not been shown in any other study so far. The prevalence of metabolic syndrome according to the IDF definition was found to be 28%, that according to Cook was found to be 38.75% and that according to De Ferranti was found to be 43.75%. The study didn’t show any significant correlation between waist circumference and elevated HOMA-IR. Among the overweight category, 83.33% (n=10) had at least one metabolic abnormality, 25% (n=3) had 2 metabolic abnormalities. The incidence of metabolic abnormalities between the obese and morbidly obese category were similar.

## Conclusion

The study shows that the prevalence of insulin resistance in the group is 26.25%, girls having higher insulin resistance than boys. 41.25% of children had hypertriglyceridemia, boys being more affected than girls. 41.25% of children also had decreased HDL, more seen among boys and those who were morbidly obese. 12.5% had both elevated triglycerides and decreased HDL. The prevalence of hypertension (8.75%) is lower than previously seen. HBA1C may be a better indicator of glucose homeostasis than fasting blood glucose alone. As insulin resistance is not one of the criterion of metabolic syndrome, 64.7% of those with insulin resistance are not included in the IDF definition of metabolic syndrome – hence a new definition including insulin resistance as one of its criterion may be required. Screening for dyslipidemia should be done even in overweight children to diagnose and prevent progression to metabolic syndrome.

